# Nanostructure sensitization of transition metal oxides for visible-light photocatalysis

**DOI:** 10.3762/bjnano.5.82

**Published:** 2014-05-23

**Authors:** Hongjun Chen, Lianzhou Wang

**Affiliations:** 1 Nanomaterials Centre, School of Chemical Engineering, The University of Queensland, St. Lucia, Brisbane, QLD, 4072, Australia

**Keywords:** carbon nanostructures, nanostructure sensitization, plasmonic metal nanostructures, quantum dots, transition metal oxides, visible-light photocatalysis

## Abstract

To better utilize the sunlight for efficient solar energy conversion, the research on visible-light active photocatalysts has recently attracted a lot of interest. The photosensitization of transition metal oxides is a promising approach for achieving effective visible-light photocatalysis. This review article primarily discusses the recent progress in the realm of a variety of nanostructured photosensitizers such as quantum dots, plasmonic metal nanostructures, and carbon nanostructures for coupling with wide-bandgap transition metal oxides to design better visible-light active photocatalysts. The underlying mechanisms of the composite photocatalysts, e.g., the light-induced charge separation and the subsequent visible-light photocatalytic reaction processes in environmental remediation and solar fuel generation fields, are also introduced. A brief outlook on the nanostructure photosensitization is also given.

## Introduction

In response to the decreasing supply of fossil fuels and the environmental problems caused by their exploitation, the search for a renewable and sustainable energy supply has been intensified in the past decades. Solar energy is clean and abundant, yet its utilization is still very low. There is a strong need for scientists to develop a sustainable and cost-effective manner for harvesting solar energy to satisfy the growing energy demand of the world with a minimal environmental impact.

**Figure 1:**
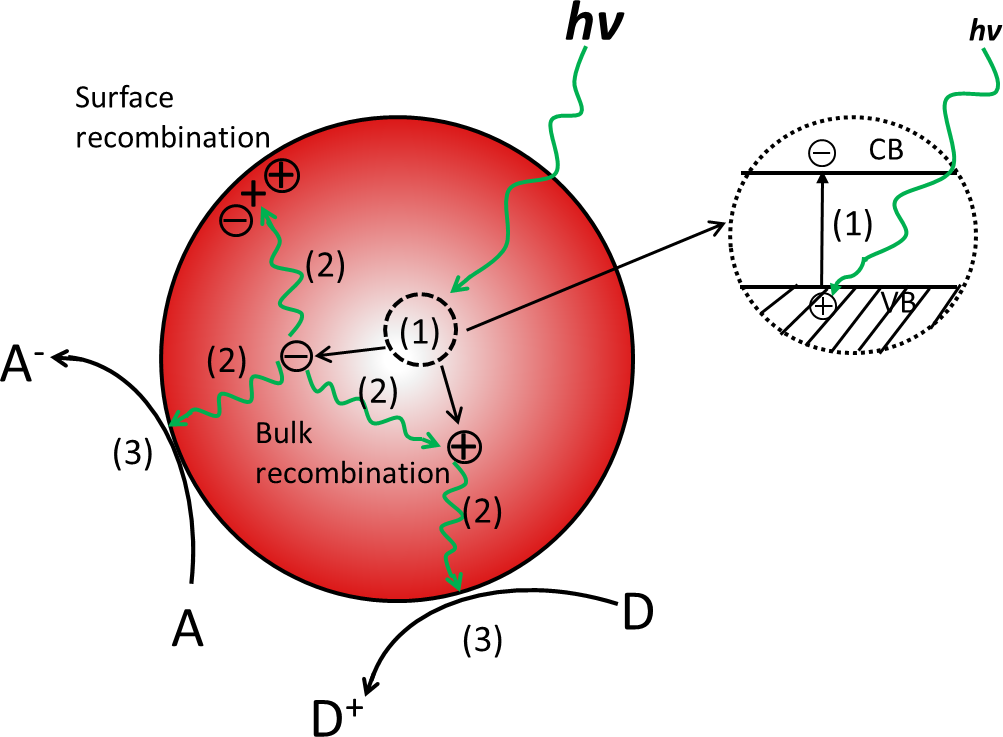
Schematic steps for the photocatalytic reactions occuring on the surface of a semiconductor. Adapted from [3].

Photocatalysis plays an important role for the conversion of solar energy into chemical fuel, electricity, the decomposition of organic pollutants etc. All of these photocatalytic reactions occur on the surface of semiconductors. Basically, the photocatalytic process can be mainly divided into three pathways [1–3], as shown in Figure 1. (1) Firstly, the incident light is absorbed by the semiconductor material, known as photocatalyst. If the incident light energy is larger than the bandgap of the photocatalyst, it can absorb light energy and further excite the electrons from the valence band (VB) to the conduction band (CB), leaving free holes in the VB. (2) The recombination of photo-generated electrons and holes (charge carriers) has frequently happened either on the surface or in the bulk of a semiconductor accompanied with releasing photons or heat. In addition to this, there is also the possibility for photo-generated electrons and holes to migrate to the surfaces of the semiconductor. (3) Subsequently, the reduction reactions happen between the photo-generated electrons and absorbed molecules on the semiconductor surface, which play the role of electron scavengers (A → A^−^). The holes can generate strong oxidizing agents like hydroxyl radicals by directly reacting with surface hydroxyl groups or oxidizing adsorbed molecules (D → D^+^). The basic mechanisms of the photocatalytic process include these reduction and oxidation reactions as well as some secondary reactions, which forms the driving force of a number of important photocatalytic applications.

Since the discovery of TiO_2_ for photoelectrochemical splitting of water in 1972 by Fujishima and Honda [4], great efforts have been directed to the research of the conversion of solar energy, in the process of which the development and utilization of a variety of semiconductor photocatalysts have received significant attention. Up to now, more than 100 photocatalysts have been developed [5–6]. However, most of the photocatalysts under investigation are wide-bandgap transition metal oxides and only active under ultraviolet (UV) light. To be of practical use for photocatalysis, the photo-response of the transition metal oxides would be required to be within the visible light spectrum. Visible light accounts for around 43% of the electromagnetic radiation on the planet’s surface compared to approximately 5% for UV light. Therefore, an appropriate photocatalyst should function in the visible-light region (420 nm < λ < 800 nm) with a band gap of less than 3 eV. An effective method used to overcome the limitation of well-developed UV-active photocatalysts is by photosensitization.

In the past two decades, the emergence of nanomaterials as new building blocks in the research area of photocatalysis has attracted increasing attention. Compared with bulk materials, nanomaterials often exhibit unusual features such as large surface areas, diverse morphologies and size-dependent physicochemical properties. Size-dependent properties include an increased absorption coefficient, increased band-gap energy, a reduced carrier-scattering rate, and higher reactive sites [7–10], which sums up to nanomaterials having superior properties in light harvesting and energy transfer efficiency. Thus, the usage of nanomaterials as new building blocks has opened a new way to utilize solar energy for the investigation of photocatalysis. There is a large array of excellent review articles covering selected aspects of the design of photocatalysts in the past years. In this review, we focus on a variety of nanostructures including quantum dots, plasmonic metal nanostructures and carbon-based nanostructures used as photosensitizers for tailoring wide-bandgap transition metal oxides toward visible light photocatalysts. A systematic overview on different nanostructured photosensitizers, light-induced charge transfer mechanisms and their potential applications is discussed in this article as well as a brief summary of the development prospects of this research topic.

## Photosensitization of transition metal oxide

Photosensitization is an effective method to improve the visible-light photocatalytic ability of wide-bandgap transition metal oxides. The photosensitized transition metal oxides can realize visible-light photocatalysis by virtue of a narrow bandgap of photosensitizers, which is fundamentally different from the metal or non-metal doped ones. The photosensitizer can be an organic dye, an inorganic complex, and different nanostructures. Normally, photosensitizers have a bandgap, which is, narrower and has a higher CB minimum or lowest unoccupied molecular orbital (LUMO) in comparison with wide-bandgap transition metal oxides. Because a photosensitizer normally has a narrow bandgap, it can absorb the visible sunlight and even the infrared sunlight to generate electron–hole pairs. Then, if coupled with a transition metal oxide, the photogenerated electrons can be easily transferred from the CB minimum of the photosensitizer or LUMO to that of a transition metal oxide. Thus the efficient charge separation in the metal oxide-photosensitizer nanocomposites facilitates visible light photocatalysis which is important for the photo-degradation of organic pollutants and the splitting of water for the production of H_2_ fuel.

In this section, we mainly focus on the different nanostructures like quantum dots, plasmonic metal nanostructures, and carbon-based nanostructures used as photosensitizers for transition metal oxides. Note that various organic dyes such as rhodamine B, porphyrins, and phthalocyanines have been employed as photosensitizers [11–14] and these dyes also play an important role in the photosensitization of dye-sensitized solar cells (DSSCs) [15–17], we will not repeatedly discuss this part. In the past decade, the rapid development of nanotechnology has provided excellent opportunities for designing a broad range of photosensitized transition metal oxide systems by using different nanostructures as the photosensitizers.

### Quantum dots as the photosensitizer

Quantum dots are fluorescent nanoparticles with sizes of several nm, which contain a core of hundreds to thousands of atoms of group II and VI elements (e.g., CdS, CdSe and CdTe) or group III and V elements (e.g., InAs and InP). Due to the quantum confinement effect of the charge carriers, quantum dots have a unique photoluminescence (PL). In comparison with organic dyes, quantum dots are characterized by unique optical and electronic properties such as a higher PL quantum efficiency, a wide continuous absorption, a narrower PL band, tunable luminescence depending only on their size, and higher photostability [8], which qualifies quantum dots as good candidates for the photosensitization of wide-band transition metal oxides.

**Figure 2:**
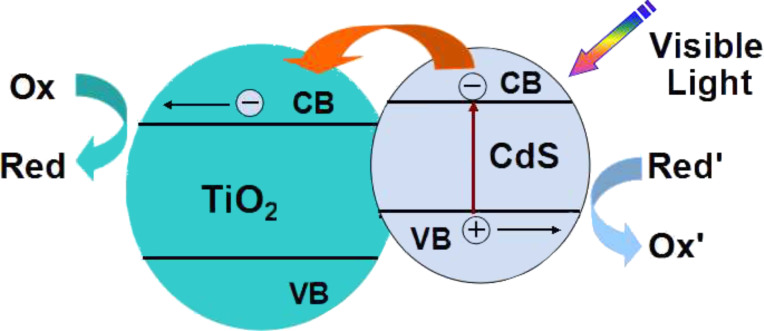
Schematic diagram illustrating the principle of charge transfer between CdS and TiO_2_.

**Figure 3:**
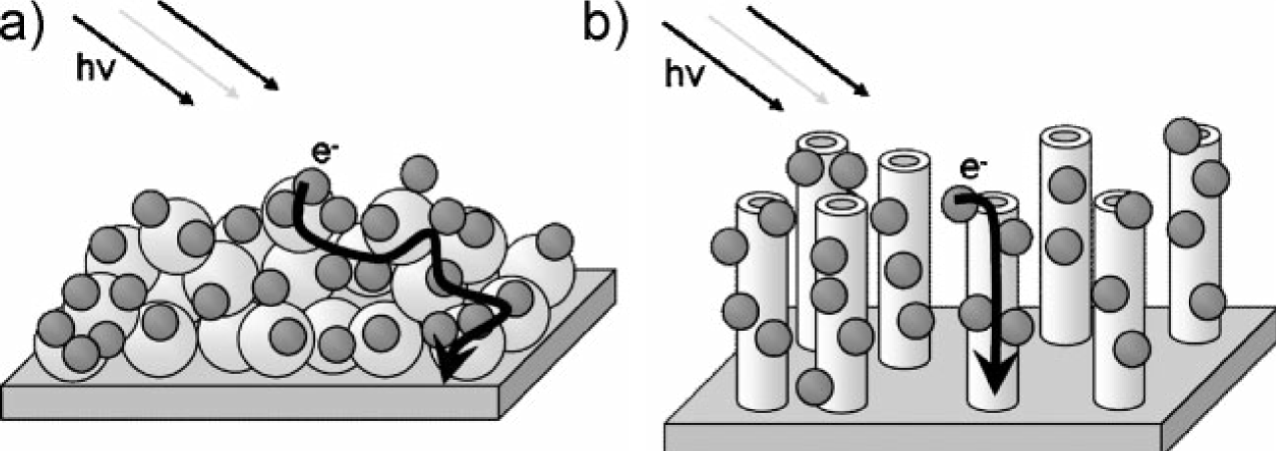
Photoinduced charge separation and transport in a) TiO_2_ particulate film and b) TiO_2_ nanotube array modified with CdS quantum dots. Reprinted from [32] copyright (2009), with permission from Wiley.

CdS is a fascinating material with ideal band gap energy (*E*_g_ = 2.4 eV) for visible light applications. As early as 1987, Spanhel et al. confirmed that CdS particles can be excited and efficiently injected electrons to the CB of the attached TiO_2_ particles under visible light illumination [18]. In the case of CdS photosensitized TiO_2_, the energy of visible light cannot directly excite TiO_2_ particle due to its wide bandgap (*E*_g_ = 3.2 eV). However, because the bandgap of CdS is much narrower, the same light source may be able to generate electron–hole pairs and excite the electrons from the VB to the CB of CdS. Because the CB of CdS is more negative than that of TiO_2_, the photogenerated electrons will transfer from the CB of CdS to the CB of the adjacent TiO_2_, while the photogenerated holes stay in the VB of CdS. Consequently, the charge separation is improved, and the separated electrons and holes are continually involved in the following reduction and oxidation reactions. The charges transfer scheme is shown in Figure 2. Zhang et al. have conducted direct femtosecond measurements of the electron transfer process from CdS to TiO_2_ and found that this process could be completed on a time constant of 2 picoseconds, resulting in a significantly slower recombination of the charge carriers generated upon light absorption in CdS [19]. Improved charge separation, decreased electron–hole recombination and enhanced photocatalytic efficiency of such photosensitized transition metal oxides attracted increasing development of CdS–metal oxide composite systems [20–26]. For example, Yu and co-workers reported a microemulsion-mediated solvothermal method for the fabrication of nanosized CdS-sensitized TiO_2_ nanocrystals [27]. The obtained photocatalysts exhibited a high efficiency for the decomposition of methylene blue under visible light irradiation, demonstrating the strong coupling and effective electron transfers between nanosized CdS and TiO_2_ nanocrystal. The formation of Ti^3+^ was observed in the electron paramagnetic resonance (EPR) spectrum, which confirms an effective transfer of photogenerated electrons from the CB of CdS to the CB of TiO_2_. The same group also successfully extended this method for the oxidation of nitric oxide in air under visible light irradiation [28]. The same configuration of CdS photosensitized TiO_2_ was also used for the photocatalytic oxidation of ethanol vapour [29] and for the production of hydrogen from aqueous H_2_S solution under visible light [30]. In addition to TiO_2_ nanocrystals, 1D tubular structure of TiO_2_ nanotubes and 2D layered titanate nanosheets were also used for the photosensitization. The CdS quantum dots photosensitized TiO_2_ nanotubes by covalent bonding [21] or a sonication-assisted sequential chemical bath deposition approach [31] led to a more efficient separation of photogenerated electrons from CdS to TiO_2_ nanotubes, and exhibited a much enhanced photocurrent generation and photocatalytic efficiency under visible-light irradiation. Kamat et al. compared the performances of CdS photosensitized ordered arrays of tubular TiO_2_ architectures with a CdS photosensitized particulate structure and found a more efficient means of separating electron–hole pairs on the tubular TiO_2_ architectures, leading to an improved performance of the CdS photosensitized tubular TiO_2_ architectures [32]. Figure 3 outlines the electron transport in these two different architectures. Kim et al. reported a layer-by-layer self-assembly between positively charged CdS quantum dots and negatively charged exfoliated titanate nanosheets to design noble-metal free photocatalysts. The resultant composites exhibited a much higher photocatalytic H_2_ production activity than pristine titanate and CdS quantum dots [33]. Such superior photocatalytic properties could be attributable to a combination of a few factors including the depression of electron–hole recombination, bandgap narrowing, and increased surface area upon hybridization.

**Figure 4:**
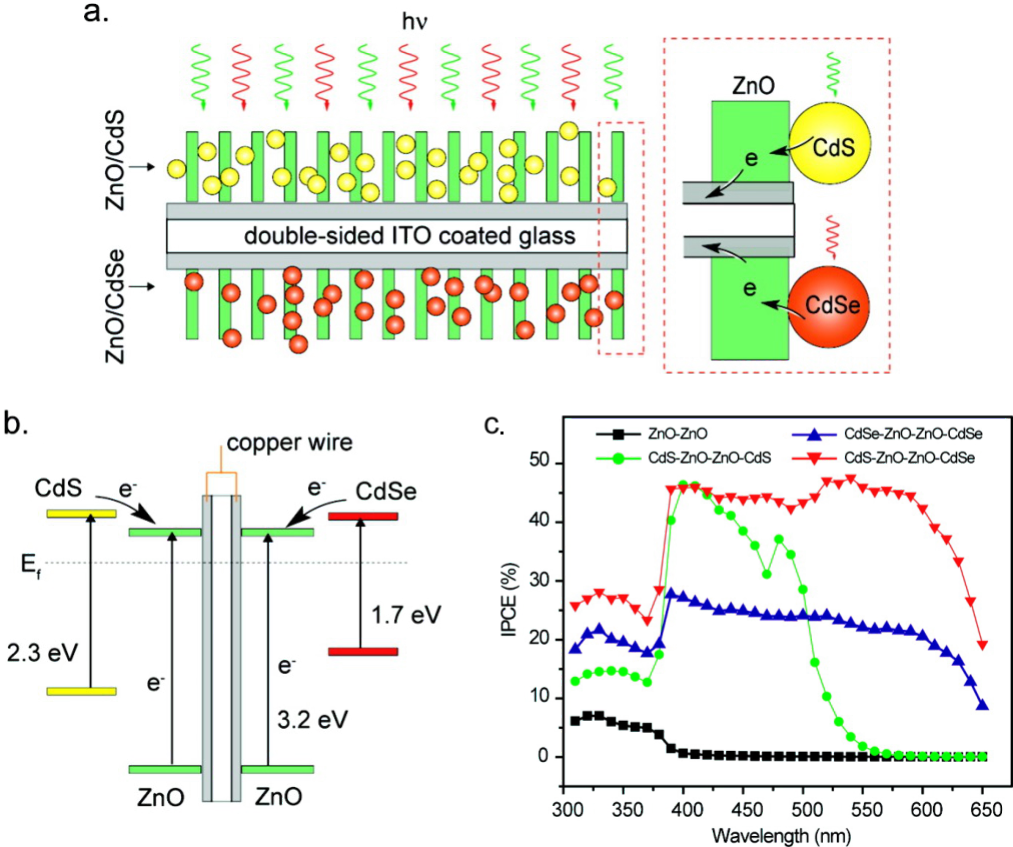
Schematic diagrams illustrating (a) the architecture and (b) the corresponding energy diagram of double-sided CdS–ZnO–ZnO–CdSe NW arrayed photoanode. The dashed box highlights the CdS–ZnO and CdSe–ZnO interfaces. (c) Measured IPCE spectra of double-sided NW samples collected at the incident wavelength range from 310 to 650 nm at a potential of 0 V vs Ag/AgCl. Reprinted from [38] copyright (2010), with permission from the American Chemical Society.

Due to the rapid development of nanotechnology, a variety of quantum dots and transition metal oxides with varied morphologies have been developed in recent years. This subsequently provides more chances for the fabrication of new classes of quantum dots photosensitized transition metal oxides. For instance, the Weller group has conducted a series of research work on the photosensitization of nanoporous titanium dioxide, zinc oxide, tin dioxide, niobium oxide, and tantalum oxide by quantum-sized cadmium sulfide, lead sulfide, silver sulfide, antimony sulfide, and bismuth sulfide. They found that the photocurrent quantum yields of these photosensitized transition metal oxides can be up to nearly 80% and open circuit voltages up to the 1 V range [24]. Ho et al. revealed that the CdSe–TiO_2_ coupled system had a much higher photocatalytic activity than that of pure TiO_2_ and CdSe in the degradation of 4-chlorophenol under visible light irradiation [34]. Wang et al. reported facet ZnO–CdS heterostructures and found that the hydrogen evolution rate over ZnO disk–CdS nanoparticle heterostructures is 2.8 times higher than the hydrogen evolution rate of the ZnO rod–CdS nanoparticle because photoexcited electron–hole separation is significantly enhanced by polar interfaces [35] and the greatly prolonged lifetime of photoexcited carriers [36]. We also investigated the photoelectrochemical behavior of CdS sensitized TiO_2_ film with {001} facet enriched anatase nanocrystals [37]. Zhang et al. developed a double-sided CdS and CdSe quantum dot co-sensitized ZnO nanowire arrayed photoanode for photoelectrochemical hydrogen generation [38]. As shown in Figure 4, the scheme shows that the quantum dots of CdS and CdSe on both sides of ITO can be excited and transfer electrons to the ZnO nanowire array. The CB edges of CdS and CdSe are higher than that of ZnO (Figure 4b). Due to this unique configuration and the band alignment between CdS and CdSe, the co-sensitized ZnO nanowire arrayed photoanode exhibited almost the entire visible-light absorption and fast electron transfer from CdSe quantum dots to ZnO nanowires and thereby the IPCE value can reach 45% at 0 V vs Ag/AgCl, as demonstrated in Figure 4c [38]. A synergetic effect of nitrogen-doping and CdSe quantum-dot-sensitization on nanocrystalline TiO_2_ was also investigated. Interestingly, a significant photoelectrochemical hydrogen generation enhancement was observed due to CdSe sensitization and N-doping that can facilitate hole transport from CdSe to TiO_2_ via oxygen vacancy states mediated by N-doping [39–40]. There are also a large number of heterostructures in literature consisting of quantum dots and transition metal oxides, for instance, CdS/CdSe co-sensitized TiO_2_ [41], CdTe or CdTe/CdSe quantum dots on TiO_2_ nanotube arrays [42–44], CdTe quantum dot monolayer sensitized ZnO nanowire [45], CdS nanoparticle/ZnO nanowire array [46–47], CdS/ TiO_2_ nanofibers heteroarchitectures [48], ZnO/CdS core/shell nanowire [49], CdS nanowires decorated with TiO_2_ nanoparticles [50], and their potential applications for photoelectrochemical water splitting in order to produce hydrogen [35,38–39,41,45] and the photocatalytic reduction of CO_2_ were reported [51]. In addition to the applications in photocatalysis, quantum dots are often used as photosensitizer to sensitize the mesoporous TiO_2_ photoelectrode as the photoanode in quantum dots solar cells. Because quantum dots solar cells are out of the topic of this review; readers may refer to recently published reviews on this topic [52–53].

The quality of heterojunctions between quantum dots and semiconductors has an important impact on the overall photoconversion efficiency. There are mainly two methods used to modify quantum dots on the surface of semiconductor. The first method is the in situ growth method. The second method is based on the ex situ assembly of pre-synthesized quantum dots by covalent bonding, electrostatic force or the external forces such as electrophoresis. If covalent bonding is used, the length of the bifunctional linker and the surface of the transition metal oxides or quantum dots all play important roles for the photosensitized charge injection. Dibbell et al. investigated the influence of bifunctional linkers with different lengths between CdS quantum dots and TiO_2_ nanoparticles on the electron injection from photoexcited CdS to TiO_2_ [54]. They found the electron injection yield decreased with increasing chain length of the linker and interparticle separation. Parkinson et al. compared the in situ ligand exchange method and the ex situ ligand exchange method for binding CdSe quantum dots on single crystal TiO_2_ surface. They revealed that the different ligand modification methods can strongly affect quantum dots adsorption and thus photosensitized charge injection [55]. Yang et al. directly compared the in situ growth method and the ex situ assembly pre-synthesized quantum dots method and found that the performance of heterojunctions by the in situ growth method exhibits a more efficient interfacial charge transfer than that of electrophoretic deposition [44]. From this comparison, it can be clearly seen that the in situ growth method can directly modify quantum dots on the surface of transition metal oxides without any linker or ligand, which is beneficial for the injection of excited charges. More importantly, the in situ growth method can load multilayer of quantum dots on the surface of transition metal oxides, which can generate many more photoexcited charges at the same time. However, the shortcoming of this method is the difficulty to control the size of the quantum dots. Due to the wide size distributions it is hard to fully utilize the size quantization effects of quantum dots. Recently, Kamat et al. highlighted a systematic evaluation of size dependent electron transfer rates in CdSe–TiO_2_ semiconductor heterostructures [56]. Based on femtosecond transient absorption measurement it was found that the CB of CdSe quantum dots become more negative and the energy difference between the CB of CdSe and TiO_2_ is much larger with decreasing particle size of CdSe quantum dots. Accordingly, the driving force and the electron transfer rate are significantly enhanced. There is still a need for the development of a novel method or a suitable adaptation of present deposition methods so that not only the size quantization effects of quantum dots are utilizable, but also an efficient interfacial charge transfer between quantum dots and semiconductors is improved upon.

The usage of quantum dots as a sensitizer is an effective method to extend the absorption of wide-bandgap semiconductor to visible light and even the IR region as well as dramatically improves their visible-light photocatalytic abilities. Yet, this method also has some inherent shortcomings. One drawback is that most quantum dots are composed of heavy metals like Cd and Pb, which gives rise to environmental concerns. The other one is the photoelectrochemical stability of quantum dots. Although quantum dots combined with a wide-bandgap transition metal oxide such as ZnO and TiO_2_ can overcome this stability issues to some extent, quantum dots still face a serious photocorrosion problem. For example, CdS is unstable due to serious self-oxidation by the photo-generated holes in the VB. In order to protect the quantum dots, a sacrificing chemical is normally required. For example, an aqueous solution of Na_2_S and Na_2_SO_3_ is normally used as an electrolyte for photoelectrochemical splitting of water. In this mixture, Na_2_S acts as a hole scavenger and is oxidized into S_2_^2−^ to prevent the photocorrosion of quantum dots, and Na_2_SO_3_ reduces S_2_^2−^ back to S^2−^ to ensure repeated usage of S^2−^. An alternative way is the fabrication of the quantum dots/transition metal oxide core/shell nanostructures. Ghows et al. reported a fast and easy way for the fabrication of CdS/TiO_2_ core/shell nanocrystal by micro-emulsion under ultrasound [57]. Co-axial arrays of CdS/TiO_2_ core/shell structures were also reported to be fabricated by an anodic aluminium oxide template [58]. This type of configuration can not only protect the quantum dots from photocorrosion, but also increases the contact interface and facilitates the vectorial electron transfer.

### Plasmonic metal nanostructures as the photosensitizer

Surface plasmon resonance (SPR) is the resonant photon-induced collective oscillation of valence electrons, which happens only if the frequency of photons matches the natural frequency of surface electrons oscillation on certain metallic nanostructures (e.g., Cu, Ag and Au). This radiation generates strong localized electromagnetic fields around the metallic nanostructures and leads to greatly enhanced optical absorption and scattering occurring at specific wavelengths, which depends not only on the nature of the metal, but also on the size and shape of the metallic nanostructures. For example, the plasmon resonance of silver can be tuned from UV to the visible range by reducing the size of silver particles in the nanometer range. Similarly, it is possible to shift the plasmon resonance of gold from the visible range to infrared wavelength by tuning the aspect ratio of different gold nanorods. Over the past years, a new method has emerged, which uses the strong plasmon resonance of plasmonic metal nanostructures for improving the efficiency of the photocatalytic process. Similar to the organic dyes or quantum dots, the plasmonic metal nanostructures can also be used as photosensitizer to effectively improve the visible-light response of transition metal oxides, which yields novel heterostructures of plasmonic metal photosensitized photocatalysts with a variety of applications including DSSC [59–63], photocatalytic water splitting [64–65] photoelectrochemical water splitting [66–72], photocatalytic conversion of CO_2_ with H_2_O to hydrocarbon fuels [73], and degradation of organic molecules [74].

**Figure 5:**
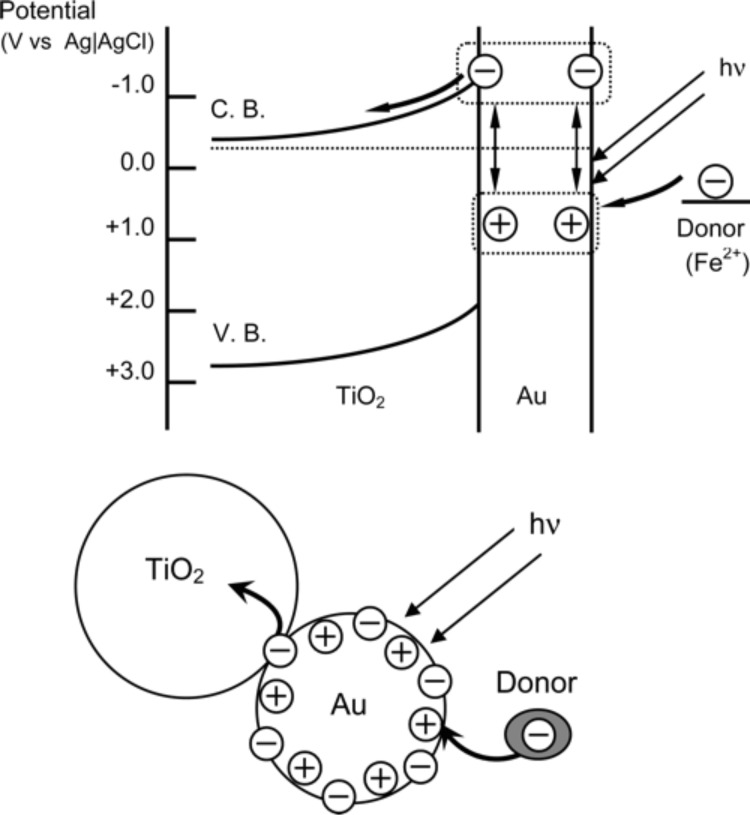
Proposed charge transfer mechanism for the visible-light-irradiated gold nanoparticle−TiO_2_ system. Reprinted from [76] copyright (2005), with permission from American Chemical Society.

In 2004, Tatsuma et al. reported that nanoporous TiO_2_ films loaded with gold and silver nanoparticles exhibited anodic photocurrents in response to visible light irradiation [75]. Based on this finding, they proposed a charge transfer mechanism to explain the phenomenon. More specifically, due to the plasmon resonance effect, gold nanoparticles can be photoexcited to generate hot electrons, which are injected from the surface of the gold nanoparticles to the CB of TiO_2_. Meanwhile, the compensative electrons can be transferred from a certain type of donor in the solution to the gold nanoparticles [76]. The proposed charge transfer mechanism is shown in Figure 5. The phenomenon of plasmon-enhanced photocatalysis was also successively discussed by other groups. For example, Primo et al. reported that 1 wt % gold-supported ceria nanoparticles generated oxygen from water under visible light (λ > 400 nm) more efficiently than the standard WO_3_ under UV irradiation [65]. Ingram et al. employed plasmonic Ag nanocubes as building blocks to fabricate silver nanocube-N doped TiO_2_ photoelectrocatalysts for PEC water splitting [67]. An investigation of the relationship between the photocurrent and light intensity by Ingram et al. revealed that N-doped TiO_2_ exhibited a half-order dependence and silver nanocube-N doped TiO_2_ displays a first-order dependence. This indicates that the intense electric fields generated at the silver nanocubes increase the formation rate of electron–hole pairs at the nearby N-doped TiO_2_ particle surface. More importantly, they also proposed a new local electric field enhancement mechanism, which is totally different from the charge transfer mechanism proposed by the Tatsuma group. Cronin and co-workers shared a viewpoint similar to that of Ingram et al. and observed enhancements of up to 66 times in the photocatalytic splitting of water in TiO_2_ nanotubes with the addition of Au nanoparticles under 633 nm illumination, but a 4-fold reduction in the photocatalytic activity under UV radiation [66]. They ascribed the improvement of visible-light photocatalytic activity to the increase of the electron–hole pair generation rate by the local electric field enhancement. After a comparison of these two different mechanisms, the main difference is the origin of the photogenerated charges. The decision which mechanism takes center stage in the plasmonic enhancement traced back to the question whether there is an overlap in the absorption spectra between transition metal oxides and gold nanoparticles. Due to N-doping and N- and F-impurities generated in the anodization process, the N-doped TiO_2_ nanoparticles and TiO_2_ nanotubes have absorption spectra in the visible range and show an overlap with that of gold nanoparticles. Therefore, when the composite photocatalysts are illuminated under visible light, the N-doped TiO_2_ nanoparticles and TiO_2_ nanotubes can generate electron–hole pairs. Meanwhile, an intense local electrical field is also generated near the surface of the gold nanoparticle due to the irradiating wavelength matching the plasmon resonance frequency of gold nanoparticles, which increases the formation rate of electron–hole pairs at the surface of the TiO_2_. Thus, the amount of photogenerated charges contributing to the visible-light photocatalysis is correspondingly increased. Consequently, the composite photocatalysts exhibit a much better photocatalytic performance than N-doped TiO_2_ nanoparticles or TiO_2_ nanotubes alone. Electromagnetic simulations based on the finite-difference time-domain method provided the theoretical support for this local electric field enhancement mechanism. In contrast to the local electric field enhancement mechanism, there is no overlap in the absorption between transition metal oxide and plasmonic metal nanostructures. Moreover, the source of the photogenerated charges is the plasmonic metal nanostructures, not the transition metal oxides.

Although two different plasmonic enhancement mechanisms are proposed, the debate is still ongoing and there are partly contradictory opinions. Therefore, many different techniques and approaches are adopted to investigate the plasmonic metal nanostructures–transition metal oxides heterostructures and unravel the mechanism behind the visible-light activity of plasmonic photocatalysts. For example, Furube et al. reported the direct evidence of plasmon-induced electron transfer from gold nanoparticles to TiO_2_ by using a femtosecond transient absorption technique. They found that the electron injection from the excited gold nanoparticles to TiO_2_ was complete within 50 fs and the electron injection yield reached 20–50% under 550 nm excitation [77–78]. Brückner et al. were first to use in situ EPR spectroscopy for monitoring water reduction over Au–TiO_2_ photocatalysts. They observed a visible-light driven electron transfer from the Au nanoparticles to the CB of TiO_2_ [79]. The results suggest that this electron transfer is a joint action by either d–sp interband transitions in the lower or SPR transitions in the higher wavelength range of the visible spectrum. Cushing et al. designed Au@SiO_2_@Cu_2_O sandwich core-shell nanostructures and used transient-absorption and photocatalysis action spectrum measurement to determine the underlying plasmonic energy-transfer mechanism [80]. Due to the insulating SiO_2_ shell the gold core can only transfer the plasmonic energy to the Cu_2_O shell by resonant energy transfer and the electron–hole pairs generated by the dipole–dipole interaction between the gold core and the Cu_2_O semiconductor shell.

**Figure 6:**
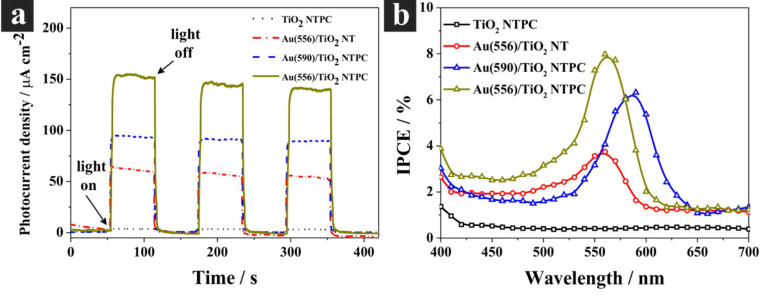
Photoelectrochemical properties of the TiO_2_ NTPC and Au/TiO_2_ NTPC and schematic diagram of SPR charge carrier transfer mechanisms. (a) Amperometric I–t curves at an applied potential of 1.23 V vs RHE under illumination of visible light with wavelength ≥420 nm and 60 s cycles of light turned on/off. (b) IPCE plots in the range of 400–700 nm at 1.23 V vs RHE. Reprinted from [72] copyright (2013), with permission from American Chemical Society.

Plasmonic metal nanostructures have been incorporated into different transition metal oxides to enhance the solar-light harvesting and the energy-conversion efficiency for the photoelectrochemical water splitting. For example, Zhang and co-workers have investigated the influence of different sizes of gold nanoparticles on the performance of the composite Au/TiO_2_ nanotube photonic crystal (NTPC) photocatalysts [72]. As described in Figure 6, a variable photocurrent density can be obtained if the TiO_2_ NTPC is modified with different sizes of gold nanoparticles under visible light illumination (≥420 nm). The gold nanoparticles with a SPR peak located at 556 nm have the maximum photocurrent density and the IPCE value can reach around 8%. The authors described the good match between the plasmonic wavelength of gold nanoparticles and the photonic bandgap of photonic crystal as the main reason for Au (556)/TiO_2_ NTPC to display the best photoelectrochemical performance among three different sizes of Au/TiO_2_ NTPC photocatalysts. There are also other groups of nanoarchitectures such as silver nanoparticle–WO_3_ [81], gold nanoparticles–Fe_2_O_3_ [69,82–83], gold nanoparticle–ZnO nanorods [68], gold nanorod–TiO_2_ [70–71,84], gold nanoparticles–TiO_2_ nanotube [66,72]. For more details, readers may refer to recent excellent reviews for basic principle and detailed effects of localized surface plasmons on transition metal oxides [85–87].

In comparison with quantum dots sensitized semiconductors the usage of plasmonic nanoparticles as photosensitizers is more stable and environmentally friendlier. The shortcoming of these plasmonic metals is their high price. This holds true for gold in particular. Moreover, the photoconversion efficiency of plasmonic nanoparticles photosensitized semiconductors is not as good as that of quantum dots sensitized semiconductors. Plasmon-enhanced photocatalysis is still in an early research stage and the underlying mechanism is unclear. Furthermore, there are contradictory phenomena reported in the literature. For example, Ingram et al. found that Au-N–TiO_2_ has no enhancement for visible-light PEC water splitting [67], while in most other cases gold nanoparticles are described as an important photosensitizer for the production of H_2_ under visible light [70–72,75–76,88]. The other disputed phenomenon is that some researchers report on the reduction of the photocurrent after the gold nanoparticles are in contact with TiO_2_ nanotubes under UV illumination [66,89], while other researchers did not observe such a phenomenon [64,72]. There are also some debates about whether the hot electrons can cross over or transfer through the potential barrier of the Schottky junction at the metal–semiconductor interface [68,88].

It is noteworthy that the plasmonic nanostructures can play different roles under UV and visible light. Under UV light, the plasmonic nanostructures play the role of a co-catalyst, which may act as electron sinks to draw them away from the holes and enhance their lifetimes [64]. Under visible light, the plasmonic nanostructures enhance the solar-light harvesting and increase the visible-light energy-conversion efficiency as photosensitizer. It is well-known that the resonant wavelength and SPR intensity depend not only on the nature of the metal, but also on the size and shape of the metallic nanostructures. The control of parameters such as composition, size and shape of plasmonic nanoparticles facilitates the design of nanostructures interacting with the entire solar spectrum [85]. Therefore, it is of great interest to develop novel classes of plasmonic nanostructures photosensitized transition metal oxides with higher photoconversion efficiency. Recently, new findings have been published. For example, Tatsuma et al. found that gold clusters can also be utilized as “organic dyes” for the conversion of light to current under visible and/or near-infrared light irradiation [90–92]. These gold clusters with tens of atoms are much smaller than gold nanoparticles, which caused gold clusters to exhibit no localized SPR but a molecular orbital. For example, Au_25_ nanoclusters are characterized by molecular-like excited-state properties with well-defined absorption and emission features, which results in Au_25_ nanoclusters acting as photosensitizer and exhibiting photoinduced electron-transfer properties. This finding may pave the way for the photosensitization of semiconductors for potential applications in solar cells and photocatalysis. Another interesting phenomenon related to plasmonic metal nanoparticles is that the metal themselve may also be used as a photocatalyst for photo-oxidation or even water splitting.

### Carbon nanostructure as the photosensitizer

**Figure 7:**
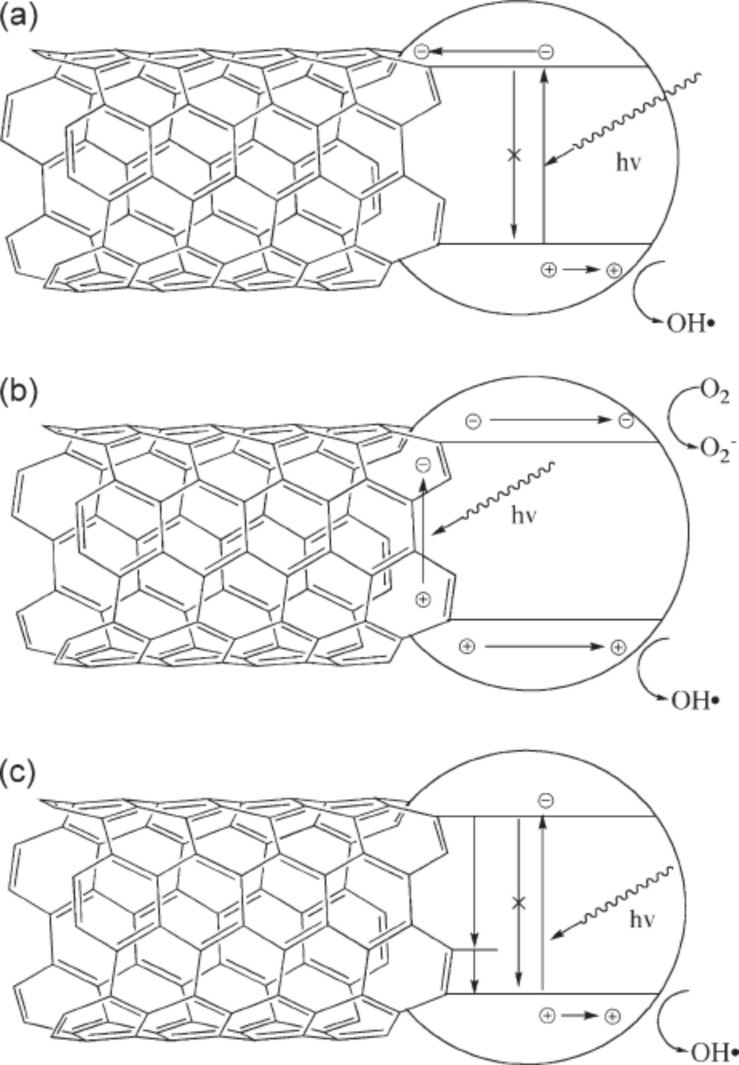
Proposed mechanisms for the carbon nanotube-mediated enhancement of photocatalysis. a) Carbon nanotubes act as electron sinks to inhibit the recombination of electron–hole pairs. b) Photosensitizing mechanism of carbon nanotubes based on generation of charge carriers in the carbon nanotubes. c) The carbon nanotubes act as an impurity by introducing additional energy levels within the transition metal oxide band gap. Reprinted from [96] copyright (2009), with permission from Wiley.

Carbon nanostructures as one of the important building blocks has been used in many research fields due to its unique properties such as good conductivity, chemical stability and high surface area. Carbon nanotube is a particular carbon nanostructure and displays a variety of unique properties such as a high number of active sites for the adsorption of reactants, good charge carrier separation and possible visible-light excitation by bandgap modification or sensitization. Consequently, carbon nanotubes are good candidates for the enhancement of photocatalysis. Usually, carbon nanotubes are used as an electron sink to improve the charge carrier separation and reduce the recombinations of electron–hole pairs. This way, the photocatalytic activity of the composite carbon nanotube/transition metal oxides is effectively improved (Figure 7a) [93]. Recently, it has been found that carbon nanostructure can also be used as a sensitizer to activate wide-bandgap semiconductors under visible light. For example, Liao et al. investigated the photocatalytic ability of the composite photocatalysts including multi-walled carbon nanotubes, TiO_2_ and Ni particle. They found that the rate of H_2_ evolution can reach 38.1 μmol/h under visible light illumination [94]. In this process, multi-walled carbon nanotube plays the role of a photosensitizer by enhancing the visible-light activity of the composite photocatalyst. The photosensitization of carbon nanotubes was also reported for carbon nanotube–TiO_2_ composite photocatalysts in the case of the photocatalytic degradation of phenol under visible light irradiation [95] (Figure 7b). In this photo-excitation process, carbon nanotubes are firstly excited by visible light and transfer electrons to the CB of a transition metal oxide for the reduction reaction to occur. Meanwhile, the positively charged carbon nanotubes extract electrons from the VB of transition metal oxide and transfer the holes to the transition metal oxide for the redox processes. Sigmund and co-workers also proposed a third mechanism as shown in Figure 7c [96], which is essentially similar to carbon-doped transition metal oxides.

**Figure 8:**
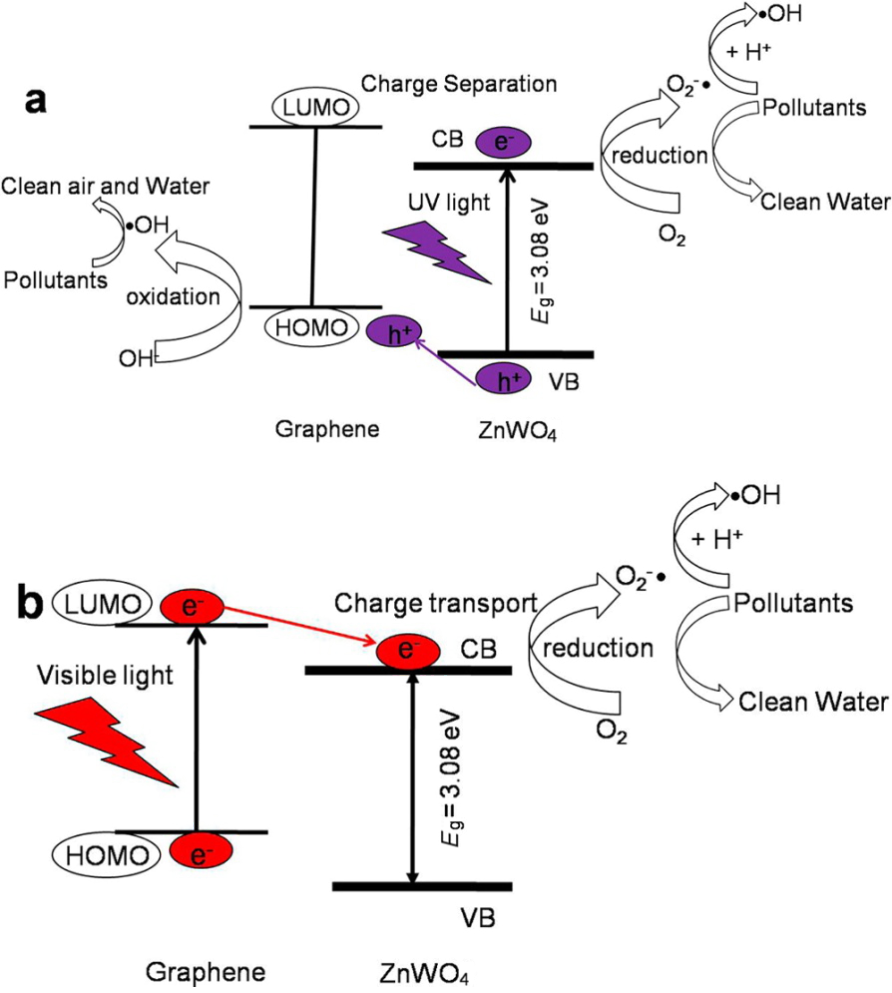
Schematic drawing illustrating the mechanism of charge separation and photocatalytic process over ZnWO_4_/graphene photocatalysts under UV light (a) and visible light (b) irradiation. Reprinted from [103] copyright (2012), with permission from the American Chemical Society.

As a representative carbon nanostructure, graphene is a 2D network of hexagonally structured sp2-hybridized carbon atoms [97]. Compared with other carbon nanostructures, graphene exhibits some outstanding properties, such as a large specific surface area (2630 m^2^ g^−1^), an exceptional conductivity (106 S cm^−1^), a fast room temperature mobility of charge carriers (200000 cm^2^ V^−1^ s^−1^), and an excellent optical transmittance (97.7%) [98]. Very recently, it has been reported that graphene has the function for the sensitization of semiconductors based on several experimental and theoretical researches. For instance, Du et al. used ab initio calculations to demonstrate that a graphene/titania interface in the ground electronic state forms a charge-transfer complex due to the large difference of work functions between graphene and TiO_2_ [99]. Most interestingly, valence electrons may be directly excited from graphene into the CB of TiO_2_ under visible light illumination, so that graphene may be used as a photosensitizer. The authors also investigated the graphene–g-C_3_N_4_ interface by hybrid functional DFT methods and found a band gap (around 70 meV) forming an electron–hole puddle in a g-C_3_N_4_-supported graphene monolayer [100]. Song and co-workers observed an enhancement of the photoconversion efficiency up to 15 times for a TiO_2_ nanotube composite electrode decorated by graphene oxide (GO) in comparison with pristine TiO_2_ nanotube arrays under identical measurement conditions [101]. The reduced graphene oxide (RGO) can act as a photosensitizer similar to organic dyes in the ZnS–RGO nanocomposites, which subsequently leads to efficient visible-light driven photoactivity for both the aerobic selective oxidation of alcohols and the epoxidation of alkenes under ambient conditions [102]. The similar photosensitization of graphene was also demonstrated in a ZnWO_4_/graphene hybrid photocatalysts for the degradation of methylene blue [103], a RGO–ZnO heterojunction for the photoelectrochemical H_2_ production [104], and GO–TiO_2_ for the photochemical water splitting [105]. The mechanisms in these research works are very similar. As shown in Figure 8a, ZnWO_4_ can absorb UV light to produce photogenerated electron–hole pairs, and then the holes transfer from the VB of ZnWO_4_ to the highest occupied molecular orbital (HOMO) of graphene because the VB position of ZnWO_4_ is lower than the HOMO of graphene. Since the CB position of ZnWO_4_ is lower than the LUMO of graphene, the photogenerated electrons can only stay at the CB of ZnWO_4_ and take part in the surface reaction to form radicals [103]. For visible light irradiation, the electrons are firstly excited from HOMO to LUMO of graphene, and then injected into the CB of ZnWO_4_ to participate in the reduction reaction on the surface, thus producing the visible-light activity (Figure 8b) [106]. This effective separation of photogenerated electron–hole pairs can effectively reduce the probability of recombination, thus resulting in an enhanced photocatalytic activity. Apart from photosensitization, graphene also has other functionalities such as the role of an electron acceptor and transporter, a cocatalyst, and a photocatalyst in the field of hydrogen generation. Readers may refer to a recently published review for more detailed information [106].

**Figure 9:**
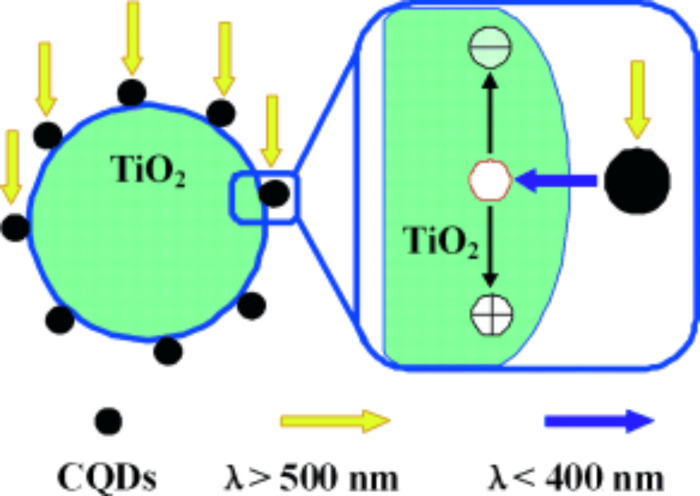
Photocatalytic mechanism for TiO_2_–carbon nanodots under visible-light illumination. Reprinted from [112] copyright (2010), with permission from Wiley.

Carbon nanodots are a new class of carbon nanomaterials and consist of discrete, quasipherical nanoparticles with sizes below 10 nm [107–110]. Since they have been reported on in 2004 for the first time [111], carbon dots have gradually become an important member in the nanocarbon family due to their benign, abundant and low-cost nature. As carbonaceous quantum dots, carbon nanodots display PL behavior dependent on their size and the excitation wavelength. In addition, carbon dots are also characterized by water solubility, chemical inertness and resistance to photobleaching. Up to now, many methods [112–127] are available for the fabrication of carbon nanodots, for instance, the electrochemical method, the microwave method, the ultrasonic method, the hydrothermal method. Due to their easy fabrication methods in addition to their unique properties carbon nanodots are a versatile material candidate for bioimaging, sensors, catalysis and photovoltaic devices [107–110]. There are also some reviews covering the synthetic methods, physical and chemical properties, and potential applications of carbon nanodots [107–110], among the latter of which is their use as a photosensitizer for the visible-light photocatalysis. For example, TiO_2_/carbon nanodots demonstrate an efficient photodegradation of methyl blue under visible light [112]. The visible-light photocatalytic mechanism for TiO_2_/carbon nanodots is schematically shown in Figure 9. When TiO_2_/carbon nanodots are illuminated by visible light, the carbon nanodots can absorb visible light (λ > 520 nm) and emit UV light (325 < λ < 425 nm) due to the PL upconversion of carbon nanodots. The UV light emitted by carbon nanodots can further excite TiO_2_ to generate electron–hole pairs, which will finally lead to the production of active oxygen radicals for the degradation of the methyl blue [112]. Besides TiO_2_/carbon nanodots, ZnO/carbon nanodots are also reported as superior photocatalysts for the degradation of benzene and methanol under visible light at room temperature [128]. The photocatalytic activities of ZnO/carbon nanodots are also reported to degrade aqueous solutions of rhodamine B under visible light irradiation [129]. Similarly, other carbon nanodots based wide-bandgap transition metal oxides, such as carbon nanodot–TiO_2_ nanotube [130], carbon nanodot–SrTiO_3_ film [131], carbon nanodot–TiO_2_ nanoparticle [114], and carbon nanodot–ZnO nanorod arrays [132], exhibited a good performance for photoelectrochemical water splitting or photocatalytic activity in dye degradation under visible light irradiation due to the photosensitization of carbon nanodots. By virtue of PL upconversion properties, carbon nanodots can also be used as photosensitizer to harness near-infrared light to further enhance the photocatalytic activity of some visible-light active semiconductors. Kang et al. recently reported that carbon nanodots can effectively harness the broad spectrum of sunlight to improve the photocatalytic activities of monoclinic bismuth vanadate (m-BiVO_4_) for the photodegradation of methylene blue [133]. The carbon nanodots play a twofold role in this photocatalytic process. Firstly, carbon nanodots function as electron collectors and transporters to trap electrons and transfer electrons generated from m-BiVO_4_ nanoparticles (λ < 520 nm), which can effectively improve the charge separation and thus the photocatalytic activity. Secondly, carbon nanodots can absorb longer wavelength light (λ > 520 nm) and then emit shorter wavelength light (300 to 530 nm) for the excitation of m-BiVO_4_ to further generate electron–hole pairs for photocatalytic degradation. Due to the special upconversion property of carbon nanodots, the carbon nanodots–m-BiVO_4_ nanospheres can be used as photocatalysts under the broad spectrum of sunshine. Based on a similar mechanism carbon nanodots can also be combined with Cu_2_O, Ag_3_PO_4_ or Fe_2_O_3_ for the photocatalytic degradation of methyl blue, methyl orange, and toxic gases of benzene and methanol, respectively [134–136].

The merits of carbon nanostructures, and carbon nanodots in particular, such as a low cost and non-toxicity, furnishes them with superior advantages compared to other photosensitizers. More importantly, their property of feature PL upconversion can even extend the activity of carbon nanodots based photocatalysts to the infrared region. Therefore, the photosensitization of carbon nanodots is effective not only for wide-bandgap semiconductors, but also for visible-light active semiconductors, so that a broad spectrum of solar energy can be efficiently used for the photocatalysis. However, it is still required to further improve the quantum efficiency of carbon nanodots based photocatalysts.

## Conclusion

**Figure 10:**
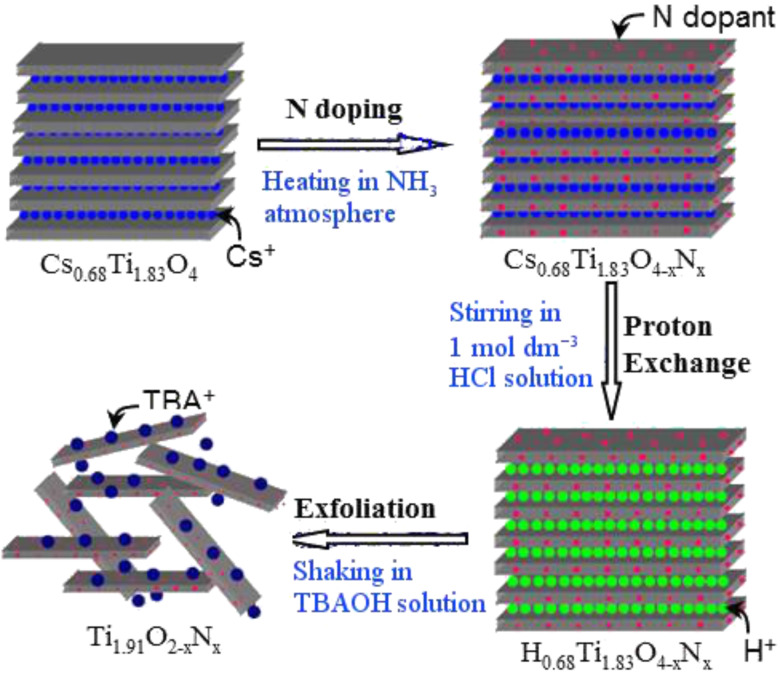
The schematic procedures for the preparation of nitrogen-doped Ti_0.91_O_2_ nanosheets. TBA^+^: tetrabutylammonium ion. Reprinted from [146] copyright (2009), with permission from the Royal Society of Chemistry.

In this review, recent advances of the nanostructure sensitization of transition metal oxides for visible-light photocatalysis are summarized. Nanostructure sensitization is an effective method to realize the transformation of UV-active transition metal oxides into visible-light-responsive photocatalysts. Thanks to the rapid development of nanotechnology, more methods are available for the synthesis of shape- and size-controlled nanostructures and facet-controlled transition metal oxides [137–139]. These advances provide a rich library for an improved photocatalyst design. For example, ion-exchangeable semiconductors, one type of transition metal oxide, have recently attracted more attention due to their inherent features. Firstly, the special gallery structures of ion-exchangeable semiconductors render them attractive for non-metal doping. We investigated a range of non-metal doping such as N, S, I and even S, N co-doping, in a serial of ion-exchangeable semiconductors [140–144]. It was found that homogeneous doping can be more easily realized on ion-exchangeable semiconductors than on bulk crystal semiconductors because the special structure of the interlayer galleries of ion-exchangeable semiconductors provides excellent channels for the diffusion of dopant and finally resulted in a uniform distribution of dopant over the whole semiconductor materials. The homogeneous doping leads to a significantly enhanced photocatalytic performance for ion-exchangeable semiconductors in the visible region. Secondly, ion-exchangeable layered semiconductors have spatially well-separated photocatalytic reduction and oxidation reaction sites, which can effectively decelerate the recombination of the photogenerated electron–hole pairs and further improve the photocatalytic ability [145]. The last feature is that an appreciable number of ion-exchangeable semiconductors can be exfoliated into single-layer two-dimensional (2D) nanosheets by the intercalation–exfoliation method, as shown in Figure 10. The thickness of the single-layer 2D nanosheets is less than 1 nm and has extremely high surface areas. Thus, exfoliated 2D nanosheets not only can be easily used as building blocks for the fabrication of photocatalysts with good photocatalytic ability [146], but also offer a nearly infinite surface area in aqueous solution for the photosensitizers to anchor. The hybridization of exfoliated nanosheets with nanosized photosensitizers often shows a tunable electronic structure and new physicochemical properties. All these features attribute to a promising future of nanostructure sensitization in the ion-exchangeable semiconductor family for photocatalytic applications.

The synergistic effect of doping and sensitization is also applied to certain wide-bandgap transition metal oxides. It is found that doping with photosensitization can dramatically change the visible-light absorption and significantly enhance the photocatalytic ability. For example, Zhang and co-workers have recently reported on the synergistic effect of CdSe quantum dot sensitization and N doping of TiO_2_ nanostructures for photoelectrochemical water splitting [39]. In this report, it has been found that the photocurrent density of CdSe/N-TiO_2_ is much larger than either TiO_2_ sensitized by CdSe quantum dots or TiO_2_ doped with N. This also demonstrates the synergistic effect in the photo-excitation process. The synergistic effect also happens on composite photocatalysts containing two different types of photosensitizers. For example, in order to extend the photocatalytic activity of a semiconductor into a much longer wavelength, two different quantum dots like CdS/CdSe or CdSe/CdTe co-sensitized ZnO or TiO_2_ have been reported [38,41,44]. The synergistic effects of CdSe quantum dots and carbon nanodots co-sensitized TiO_2_ for the photoelectrochemcial hydrogen generation has also been explored [147]. In this case, carbon nanodots absorb near-infrared photons (λ > 750 nm) and emit visible photons through the upconversion effect to excite CdSe again. The strategy of these studies may pave the way for the design of near-infrared active photoelectrochemical systems in the future.

There are also new nanostructures with special features for the potential application in visible-light photocatalysis. For example, very recently, Heinz and co-workers found that by decreasing the thickness of MoS_2_ to a monolayer [148], the indirect bandgap bulk semiconductor can change into a direct bandgap. Correspondingly, the monolayer of MoS_2_ exhibits 104 times enhancement of luminescence quantum efficiency than that of bulk material. This new finding has also been verified by other research groups [149–150]. Based on this new finding, one may easily imagine that the monolayer of MoS_2_ has great potential as a photosensitizer in the near future. In addition to monolayer MoS_2_, the nanostructures of gold clusters and carbon nanodots are still in a stage of early development, providing numerous challenges and opportunities for future investigation.

Although visible-light photocatalysis is still in an early phase of development, the photocatalysts have played effective roles in many potential applications including solar fuel production, pollutant decomposition, and water/air purification. In the course of, we believe that a fundamental understanding of the photocatalytic process and the rational design of visible-light photocatalysts with a high conversion efficiency may lead to an important role of nanostructure photosensitization in visible-light photocatalysis for efficient solar energy conversion.
